# A Scoping Review of Aging in Place: Is the Current Literature Inclusive of African American Women?

**DOI:** 10.1093/geroni/igaa057.158

**Published:** 2020-12-16

**Authors:** Reginald Tucker-Seeley, Carlene Davis, Jubilee Ahazie, Matti Robi, Leora Steinberg

**Affiliations:** 1 University of Southern California, Los Angeles, California, United States; 2 Sistahs Aging with Grace & Elegance, Los Angeles, California, United States; 3 University of Southern Califonia, Los Angeles, California, United States

## Abstract

Approximately 80% of adults aged 50+ report a desire to stay in their homes as long as possible, or to “age in place.” Yet, as the US aging population becomes more racially and ethnically diverse, the frameworks used to describe “aging in place” will require explicit recognition of the issues specific to racial/ethnic minorities. For example, given the intersection of historical discrimination related to race, gender, age, and lower socioeconomic status, older African American women are at increased risk for poor health outcomes as they attempt to “age in place.” We conducted a scoping review of “aging in place” studies in the US (N=479), to determine whether current “aging in place” frameworks included issues relevant to racial/ethnic minorities generally, and African American women, specifically. Our inclusion criteria for articles were as follows: 1) a definition of “aging in place”; 2) a conceptual framework related to “aging in place” and/or 3) the incorporation of racial/ethnic minorities generally and African-American women specifically in the research sample. We adapted the PRISMA (Preferred Reporting Items for Systematic reviews and Meta-Analyses) framework for our review to search the PubMed database for “aging in place” studies. After applying our inclusion/exclusion criteria (N=244), the findings from our review showed that approximately 40% of studies included an explicit definition of “aging in place” (N=100), but few studies focused on African American women (N=20). Future studies on “aging in place” should consider the unique challenges that African American women face as they navigate the challenges of “aging in place.”

